# Induction of Glutathione Synthesis and Glutathione Reductase Activity by Abiotic Stresses in Maize and Wheat

**DOI:** 10.1100/tsw.2002.812

**Published:** 2002-06-21

**Authors:** Gábor Kocsy, Gabriella Szalai, Gabor Gáliba

**Affiliations:** Agricultural Research Institute of the Hungarian Academy of Sciences, H-2462, Martonvásár, Hungary

**Keywords:** stress, glutathione, maize, wheat

## Abstract

The effect of different abiotic stresses (extreme temperatures and osmotic stress) on the synthesis of glutathione and hydroxymethylglutathione, on the ratio of the reduced to oxidised forms of these thiols (GSH/GSSG, hmGSH/hmGSSG), and on the glutathione reductase (GR) activity was studied in maize and wheat genotypes having different sensitivity to low temperature stress. Cold treatment induced a greater increase in total glutathione (TG) content and in GR activity in tolerant genotypes of both species than in sensitive ones. The GSH/GSSG and hmGSH/hmGSSG ratios were increased by this treatment only in the frost-tolerant wheat variety. High-temperature stress increased the TG content and the GSH/GSSG ratio only in the chilling-sensitive maize genotype, but GR activity was greater after this treatment in both maize genotypes. Osmotic stress resulted in a great increase in the TG content in wheat and the GR activity in maize. The amount of total hydroxymethylglutathione increased following all stress treatments. These results indicate the involvement of these antioxidants in the stress responses of wheat and maize.

**ABBREVIATIONS:** APS – adenosine 5'-phosphosulfate, CS – *Triticum aestivum* L. cv. Chinese Spring, Ch – *T. aestivum* L. cv. Cheyenne, GR – glutathione reductase, GSH – reduced glutathione, GSSG – oxidised glutathione, hmGSH – reduced hydroxymethylglutathione, Pen – *Zea mays* L. cv. Penjalinan, PEG – polyethylene glycol, TC – total cysteine, TEC – total γ-glutamylcysteine, TG – total glutathione, THMG – total hydroxymethylglutathione

## INTRODUCTION

Various abiotic stresses induce oxidative stress, which arises due to the imbalance between the production and removal of reactive oxygen species, an excess of which may damage the nucleic acids, proteins, membrane lipids, and pigments. Besides their disadvantageous effects, reactive oxygen species also have positive functions in living organisms because of their involvement in the activation of various protective mechanisms through a redox signalling pathway[1,2]. The antioxidative system plays an important role in controlling the level of reactive oxygen species. The ascorbate-glutathione cycle, which is part of this system, removes excess hydrogen peroxide. Glutathione, an important component of this system, is synthesized in two steps: first, cysteine and glutamate are bound to γ-glutamylcysteine (γEC) by γEC synthetase (EC 6.3.2.2), then a glycine is added to the dipeptide by glutathione synthetase (EC 6.3.2.3). During $H_2O_2$ degradation, the reduced form of glutathione (GSH, γ-Glu-Cys-Gly) is converted to its oxidised form (GSSG) and GSH is regenerated by glutathione reductase (GR, EC 1.6.4.2)[1,3]. In wheat a homologue of GSH, hydroxymethylglutathione, is also present, in which glycine is replaced by serine[4].

Under natural conditions, the TG content increased in white pine during winter[5] and in alpine plants with increasing altitude[6]. In growth chambers, low temperature resulted in TG accumulation in maize[7]. The activity of GR, which ensures an appropriate GSH/GSSG ratio for the efficient function of the ascorbate-glutathione cycle, was greater during winter compared to summer in spruce[8]. Similarly to low-temperature stress, heat stress also increased the TG content in maize[9]. Greater GR activity was observed after heat stress in mustard seedlings[10]. Not only extreme temperatures, but withholding of water also increased the TG content, as was observed in leaves of wheat[11]. Polyethylene-glycol–induced osmotic stress increased the GR activity in wheat[12]. These results show that both GSH accumulation and GR activity are induced by different abiotic stresses.

The aim of the present study was to compare the effects of high and low temperatures and osmotic stress on GSH synthesis and GR activity in maize and wheat genotypes with different sensitivities to low temperature stress.

## MATERIALS AND METHODS

Seeds of a chilling-sensitive maize (*Zea mays* L.) line, Penjalinan (Pen), originating from the subtropical region, and a chilling-tolerant one, Z 7, originating from the temperate zone, as well as seeds of a frost-sensitive spring wheat (*Triticum aestivum* L.) variety, Chinese Spring (CS), and a frost-tolerant winter one, Cheyenne (Ch), were germinated between wet filter papers for 3 days in the dark. The maize and wheat seedlings were grown until the complete development of the second leaves in growth chambers on half-strength Hoagland solution at 25°C, 300 µmol photons $m^{-2}$ $s^{-1}$ with a 12-h photoperiod and at 15/10°C, 260 µmol photons $m^{-2}$ $s^{-1}$ with a 16-h photoperiod, respectively. In the various treatments the maize seedlings were further cultivated under the control conditions for 7 days, at 5°C for 7 days, at 25°C for 6 days and subsequently at 40°C for 1 day, or on a medium containing 11% (w/v) polyethylene glycol (PEG, osmotic pressure 0.25 MPa) at 25°C for 7 days. The wheat seedlings were cultivated for an additional 3 days at 15/10°C, at 2°C, at 37°C, or on a medium containing 11% PEG at 15/10°C.

Total thiol contents were determined after reduction with $NaBH_4$, derivatization with monobromobimane and separation by reverse-phase HPLC using fluorescence detection[13]. The following retention times were detected: cysteine – 7.2 min, γEC – 9.8 min, hmGSH – 10.3 min, and GSH – 11.8 min. The recovery of the individual thiols varied between 90 and 95%. The measurement of GR activity was based on the reaction of GSH with 5,5`-dithiobis(2-nitrobenzoic acid). The yellow product of this reaction was determined with a spectrophotometer, as described elsewhere[13].

## RESULTS

In maize, the total cysteine (TC) content was similar in the two genotypes under control conditions (Table 1). Chilling induced a great increase in the TC content in the chilling-tolerant maize genotype Z 7, while heat stress resulted in greater TC content in the chilling-sensitive Pen. Polyethylene glycol (PEG) treatment decreased the TC level in both maize genotypes. Without stress treatments, the total γ-glutamylcysteine (TEC) level was 40% greater in the tolerant Z 7 compared to the sensitive Pen. Low-temperature stress reduced TEC content in both genotypes, and heat stress increased it. Under control conditions, the TG content was lower in the chilling-sensitive Pen compared to the chilling-tolerant Z 7. Chilling resulted in a much greater increase in TG in Z 7 than in Pen. Heat stress had the opposite effect. PEG treatment reduced the TG content in both genotypes. Although without stress treatment the GSH/GSSG ratio was greater in the chilling-tolerant Z 7 compared to the chilling-sensitive Pen, a similar difference in the GR activity was not found (Table 2). Chilling decreased the GSH/GSSG ratio in both genotypes. Heat and osmotic stresses decreased the GSH/GSSG ratio in Z 7. In Pen, heat stress resulted in a greater GSH/GSSG ratio, but this was not influenced by osmotic stress. GR activity was increased by all the abiotic stress treatments in both genotypes.

In wheat, the TC content was similar in both genotypes under control conditions (Table 3). Cold hardening resulted in its greater increase in the frost-tolerant Ch compared to the frost-sensitive CS. After heat stress, the TC content was greater in CS than in Ch. PEG treatment increased the TC level only in CS. The TEC content was greater both under control conditions and after cold hardening in the tolerant Ch. Heat stress decreased TEC in Ch and increased it in CS. PEG treatment reduced the TEC content in both genotypes. The THMG level was similar under control conditions, and increased after all stress treatments in both wheat genotypes, the greatest level being detected after PEG treatment. The TG content was not significantly different in the two wheat genotypes under control conditions. Low-temperature and osmotic stress resulted in a greater increase in the tolerant Ch than in the sensitive CS. After heat stress, TG

**TABLE 1**
**Effect of Abiotic Stresses on Total Cysteine, Total γ-Glutamylcysteine, and Total Glutathione Content in Shoots of a Chilling-Tolerant Maize Line, Z 7, and a Chilling-Sensitive Penjalinan**

| | TC [nmol (g FW)$^{-1}$] | | TEC [μkat (g prot.)$^{-1}$] | | TG [nmol (g FW)$^{-1}$] | |
|---|---|---|---|---|---|---|
| | Z 7 (tol) | Pen (sen) | Z 7 (tol) | Pen (sen) | Z 7 (tol) | Pen (sen) |
| C | 33.9 ± 3.5$^{a}$ | 30.1 ± 2.8$^{ac}$ | 10.1 ± 1.2$^{a}$ | 5.8 ± 0.6$^{c}$ | 146.1 ± 17.4$^{a}$ | 115.1 ± 12.6$^{c}$ |
| 5°C | 46.1 ± 4.1$^{b}$ | 17.3 ± 1.5$^{d}$ | 7.7 ± 0.6$^{b}$ | 4.5 ± 0.5$^{d}$ | 285.5 ± 32.1$^{b}$ | 139.7 ± 11.9$^{a}$ |
| 40°C | 26.5 ± 2.3$^{c}$ | 101.2 ± 9.2$^{e}$ | 12.2 ± 1.1$^{ae}$ | 14.1 ± 1.2$^{e}$ | 161.9 ± 12.4$^{a}$ | 176.1 ± 18.2$^{a}$ |
| PEG | 14.8 ± 1.7$^{d}$ | 22.6 ± 2.1$^{c}$ | 8.3 ± 0.9$^{ab}$ | 6.2 ± 0.8$^{c}$ | 117.5 ± 10.9$^{c}$ | 89.8 ± 8.3$^{d}$ |

*Note:* C, control; PEG, polyethylene glycol; Pen, maize line Penjalinan; sen, chilling-sensitive; TC, total cysteine; TEC, total γ-glutamylcysteine; TG, total glutathione; tol, chilling-tolerant; Z 7, maize line Z 7.

After the complete development of the second leaves, the 10-day-old seedlings were subjected to the following treatments: further 7-day cultivation at 25°C (control), chilling at 5°C for 7 days, cultivation at 25°C for 6 days and subsequently at 40°C for 1 day, or cultivation on a medium containing 11% (w/v) polyethylene glycol at 25°C for 7 days. Mean values ±SD of six measurements from three independent experiments are presented. Values carrying different letters are significantly different at the $p < 5\%$ level.

**TABLE 2**
**Effect of Abiotic Stresses on the Ratio of Reduced to Oxidised Glutathione and on the Glutathione Reductase Activity in Shoots of a Chilling-Tolerant Maize Line, Z 7, and a Chilling-Sensitive Penjalinan**

| | GSH/GSSG | | GR [μkat (g prot.)$^{-1}$] | |
|---|---|---|---|---|
| | **Z 7 (tol)** | **Pen (sen)** | **Z 7 (tol)** | **Pen (sen)** |
| C | 7.4 ± 0.7$^{a}$ | 5.6 ± 0.7$^{b}$ | 0.51 ± 0.06$^{a}$ | 0.57 ± 0.07$^{a}$ |
| 5°C | 6.1 ± 0.5$^{b}$ | 2.9 ± 0.2$^{d}$ | 1.35 ± 0.12$^{b}$ | 1.07 ± 0.09$^{d}$ |
| 40°C | 4.4 ± 0.3$^{c}$ | 7.2 ± 0.5$^{a}$ | 0.85 ± 0.09$^{c}$ | 0.75 ± 0.05$^{c}$ |
| PEG | 3.3 ± 0.3$^{d}$ | 6.4 ± 0.6$^{ab}$ | 1.16 ± 0.11$^{bd}$ | 0.88 ± 0.09$^{c}$ |

*Note:* C, control; GR, glutathione reductase; GSH, reduced glutathione; GSSG, oxidised glutatione; PEG, polyethylene glycol; Pen, maize line Penjalinan; sen, chilling-sensitive; tol, chilling-tolerant; Z 7, maize line Z 7.

After the complete development of the second leaves, the 10-day-old seedlings were subjected to the following treatments: further 7-day cultivation at 25°C (control), chilling at 5°C for 7 days, cultivation at 25°C for 6 days and subsequently at 40°C for 1 day, or cultivation on a medium containing 11% (w/v) polyethylene glycol at 25°C for 7 days. Mean values ±SD of six measurements from three independent experiments are presented. Values carrying different letters are significantly different at the $p < 5\%$ level.

**TABLE 3**
**Effect of Abiotic Stresses on Total Cysteine, Total γ-Glutamylcysteine, Total Hydroxymethylglutathione, and Total Glutathione Content in Shoots of a Frost-Tolerant Wheat Variety, Cheyenne (Ch), and a Frost-Sensitive Chinese Spring**

| | TC [nmol (g FW)$^{-1}$] | | TEC [nmol (g FW)$^{-1}$] | |
|---|---|---|---|---|
| | **Ch (tol)** | **CS (sen)** | **Ch (tol)** | **CS (sen)** |
| C | 33.4 ± 3.1$^{a}$ | 31.6 ± 2.9$^{a}$ | 10.2 ± 0.9$^{a}$ | 8.4 ± 0.6$^{b}$ |
| 5°C | 68.3 ± 5.3$^{b}$ | 45.4 ± 6.4$^{c}$ | 8.3 ± 0.7$^{b}$ | 6.1 ± 0.3$^{cd}$ |
| 37°C | 43.3 ± 3.8$^{c}$ | 52.8 ± 4.8$^{d}$ | 6.9 ± 0.5$^{c}$ | 12.8 ± 1.1$^{a}$ |
| PEG | 38.6 ± 2.8$^{ac}$ | 42.7 ± 3.7$^{c}$ | 5.7 ± 0.4$^{d}$ | 5.1 ± 0.4$^{d}$ |
| | **THMG [nmol (g FW)$^{-1}$]** | | **TG [nmol (g FW)$^{-1}$]** | |
| | **Ch (tol)** | **CS (sen)** | **Ch (tol)** | **CS (sen)** |
| C | 78.1 ± 6.2$^{a}$ | 69.3 ± 6.4$^{a}$ | 458.3 ± 52.6$^{a}$ | 426.9 ± 38.6$^{a}$ |
| 5°C | 123.4 ± 14.5$^{b}$ | 112.6 ± 12.5$^{b}$ | 745.6 ± 71.4$^{b}$ | 612.4 ± 2.3$^{e}$ |
| 37°C | 105.2 ± 9.1$^{b}$ | 124.5 ± 13.7$^{b}$ | 185.7 ± 19.2$^{c}$ | 172.4 ± 17.4$^{c}$ |
| PEG | 148.9 ± 15.4$^{bc}$ | 168.5 ± 15.9$^{c}$ | 951.4 ± 89.5$^{d}$ | 657.1 ± 62.3$^{b}$ |

*Note:* C, control; Ch, wheat variety Cheyenne; CS, wheat variety Chinese Spring; PEG, polyethylene glycol; sen, frost-sensitive; TC, total cysteine; TEC, total γglutamylcysteine; TG, total glutathione; THMG, total hydroxymethylglutathione; tol, frost-tolerant.

After the complete development of the second leaves, the 10-day-old seedlings were subjected to the following treatments: further 3-day cultivation at 15/10°C (control), at 2°C (cold hardening), at 37°C (heat stress), or on a medium containing 11% (w/v) polyethylene glycol at 15/10°C. Mean values ±SD of six measurements from three independent experiments are presented. Values carrying different letters are significantly different at the $p < 5\%$ level.

decreased to similar levels in both genotypes. Neither the hmGSH/hmGSSG and GSH/GSSG ratios nor the GR activity differed in the two wheat genotypes under control conditions (Table 4). The hmGSH/hmGSSG and GSH/GSSG ratios increased only after cold hardening in Ch. After heat stress, these ratios and GR activity were greater in the sensitive CS compared to the tolerant Ch. GR activity increased after cold treatment only in Ch; following heat stress it decreased and after osmotic stress it remained unchanged in both genotypes.

**TABLE 4**
**Effect of Abiotic Stresses on the Ratio of Reduced to Oxidised Hydroxymethylglutathione and Glutathione and on Glutathione Reductase Activity in Shoots of a Frost-Tolerant Wheat Variety, Cheyenne (Ch), and a Frost-Sensitive Chinese Spring**

| | hmGSH/hmGSSG | | GSH/GSSG | | GR [μkat (g prot.)$^{-1}$] | |
|---|---|---|---|---|---|---|
| | **Ch (tol)** | **CS (sen)** | **Ch (tol)** | **CS (sen)** | **Ch (tol)** | **CS (sen)** |
| Control | 5.7 ± 0.6[a] | 6.1 ± 0.5[a] | 7.2 ± 0.8[a] | 8.3 ± 0.9[ab] | 3.22 ± 0.35[a] | 2.92 ± 0.32[a] |
| 5°C | 8.8 ± 0.7[b] | 3.4 ± 0.4[c] | 9.3 ± 1.1[b] | 5.1 ± 0.6[e] | 5.51 ± 0.61[b] | 3.43 ± 0.38[a] |
| 40°C | 2.8 ± 0.2[c] | 5.4 ± 0.7[a] | 2.4 ± 0.1[c] | 4.9 ± 0.5[e] | 1.91 ± 0.15[c] | 2.36 ± 0.24[d] |
| 11% PEG | 2.6 ± 0.3[c] | 3.1 ± 0.2[c] | 3.6 ± 0.4[d] | 3.7 ± 0.3[d] | 3.07 ± 0.34[a] | 3.75 ± 0.41[a] |

*Note:* C, control; Ch, wheat variety Cheyenne; CS, wheat variety Chinese Spring; GR, glutathione reductase; GSH, reduced glutathione; GSSG, oxidised glutathione; hmGSH, reduced hydroxymethylglutathione; hmGSSG, oxidised hydroxymethylglutathione; PEG, polyethylene glycol; sen, frost-sensitive; tol, frost-tolerant.

After the complete development of the second leaves, the 10-day-old seedlings were subjected to the following treatments: further 3-day cultivation at 15/10°C (control), at 2°C (cold hardening), at 37°C (heat stress) or on a medium containing 11% (w/v) polyethylene glycol at 15/10°C. Mean values ±SD of six measurements from three independent experiments are presented. Values carrying different letters are significantly different at the $p < 5\%$ level.

## DISCUSSION

The present results support the hypothesis that GSH is involved in the response to low temperature stress, since both in maize and wheat its synthesis was induced to a greater extent in tolerant genotypes than in the sensitive ones. The higher cysteine levels in these genotypes made increased GSH synthesis possible. In a previous study on substitution lines of the same two wheat genotypes it was shown that in the CS genetic background the Ch 5A chromosome (which carries the gene *Fr1* affecting frost tolerance[14]) increased not only the frost tolerance but the GSH levels as well[15]. This phenomenon suggests the possible involvement of GSH biosynthesis in the development of frost hardiness in wheat. The greater GSH/GSSG and hmGSH/hmGSSG ratio in the frost-tolerant wheat genotype can be explained by the greater activity of GR, since this enzyme uses both thiols as substrate. Similarly to the present observations in maize and wheat, cold treatment resulted in a greater increase in TG in chilling-tolerant genotype of tomato[16] compared to the sensitive one. The relationship between the increased TG level, GR activity and stress tolerance was also proved by chemical manipulation of their levels and using transgenic plants. The induction of GSH synthesis and GR activity by herbicide safeners increased the chilling tolerance of the chilling-sensitive maize genotype Pen[17]. The over-expression of GR in the chloroplasts of poplar resulted in increased antioxidative capacity and lower sensitivity to high light and cold treatment[18].

Heat stress resulted in a 28% greater increase in TG in the chilling-sensitive maize genotype compared to the sensitive one and in a 25% greater increase in THMG in the frost-sensitive wheat genotype. This difference between the genotypes examined was due to the greater induction of cysteine synthesis in the sensitive ones. In the case of maize the origin of the two lines (Pen – subtropical, Z 7 – temperate zone) may explain the chilling sensitivity-dependent changes in TG and in the GSH/GSSG ratio both at high and low temperatures. In wheat a similar tendency was observed for the hmGSH/hmGSSG and GSH/GSSG ratios as well as for GR activity, which parameters were greater after cold treatment in the frost-tolerant Ch and after heat stress in the frost-sensitive CS. As in the chilling-sensitive maize line, heat stress also increased the TG level and GR activity in mustard seedlings[10]. In agreement with the results obtained here in bread wheat, the GSH/GSSG ratio also decreased in heat-stressed durum wheat[19].

The reduced TG content in maize subjected to osmotic stress can be explained by the reduced availability of cysteine. Contrary to maize, higher cysteine synthesis was found in both wheat genotypes during osmotic stress, which led to increased THMG and TG levels. While a greater GSH/GSSG ratio was detected in the chilling-sensitive maize genotype after PEG treatment, a similar difference was not observed between the wheat genotypes examined. As observed in maize, osmotic stress also decreased TG in pea[20]. In the present experiment osmotic stress induced by PEG decreased the GSH/GSSG ratio in bread wheat, but the withholding of water increased it in durum wheat[20]. The present results regarding the GR activity in wheat seem to contradict those of Baisak et al.[12], who found an increase in GR activity after PEG treatment in wheat. These authors, however, used leaf segments for the experiments instead of intact plants.

The increase in TG or THMG content after various abiotic stress treatments could be a result of the increased activity of adenosine 5'-phosphosulfate (APS) reductase, the regulatory enzyme of sulfate assimilation, as described for chilled maize[21], since cysteine, the precursor of GSH, is formed in this pathway. Besides this enzyme cold treatment also increased the activity of γ-glutamylcysteine synthetase, the regulatory enzyme of GSH synthesis[21]. The difference between the TG accumulation in the maize and wheat genotypes in the present study could be due to the different activation of these enzymes by the stress treatments applied, as found in the investigation of *de novo* GSH synthesis using [$^{35}$S]sulfate in maize and wheat subjected to low temperature stress[15,21]. In these experiments a higher level of $^{35}$S-labelled cysteine and GSH was found after cold treatment in the chilling-tolerant maize and the frost-tolerant wheat seedlings than in the sensitive ones. As described for cysteine, cold treatment in maize also induced the accumulation of glutamate and glycine, the other two precursors of GSH synthesis, which may also contribute to the increased GSH synthesis[22]. Besides the increased GSH synthesis, its reduced degradation may also contribute to the stress-induced GSH accumulation.

## CONCLUSIONS

1. Although all the abiotic stresses studied induced oxidative stress, the alterations in TG and THMG levels, in the GSH/GSSG and hmGSH/hmGSSG ratios, and in the GR activity differed in response to low and high temperature and osmotic stresses in maize and wheat.
2. The cold-induced changes in TG content, GSH/GSSG ratio, and GR activity depended on the chilling tolerance of the maize and on the frost tolerance of the wheat genotypes. After heat and osmotic stresses such a relationship was only found in a few instances (i.e., heat: maize – TG and GSH/GSSG, wheat – GR, hmGSH/hmGSSG and GSH/GSSG; PEG: maize – TG and GR; wheat – TG).

## ACKNOWLEDGEMENTS

Thanks are due to L. Stéhli, M. Csollány, and A. Horváth for their technical work, to B. Harasztos for correcting the manuscript linguistically, and to J. Sutka (Agr. Res. Inst., Martonvásár, Hungary) and P. Stamp (IPW, ETH Zürich, Switzerland) for providing the seed material of wheat and maize, respectively. This work was supported by the Hungarian Scientific Research Fund (OTKA T037280, F026236 and M28074), and the Hungarian Ministry of Education (Széchenyi Programme – NFKP 4/038/2001, OMFB-02579/2000).
